# Effect of Ion Size on Pressure-Induced Infiltration of a Zeolite-Based Nanofluidic System

**DOI:** 10.3390/molecules28166013

**Published:** 2023-08-11

**Authors:** Yafei Zhang, Haitao Wang, Rui Luo, Yihua Dou

**Affiliations:** 1 College of Mechanical Engineering, Xi’an Shiyou University, Xi’an 710065, China; 2 Xi’an Key Laboratory of Integrity Evaluation of Highly Difficult and Complex Oil and Gas Wells, Xi’an 710065, China; 3 Xi’an Thermal Power Research Institute Co., Ltd., Xi’an 710032, China

**Keywords:** pressure-induced infiltration, nanofluidic system, ion size, zeolite

## Abstract

A nanofluidic system consists of a nano-porous medium and functional liquid, which demonstrates a higher energy absorption density compared to conventional systems for energy absorption. Alterations in the composition of the functional liquid can significantly impact the properties of a nanofluidic system. In this paper, the widely used zeolite ZSM-5 was chosen as the porous medium to establish a nanofluidic system. Three distinct electrolyte solutions, namely KCl aqueous solutions, NaCl aqueous solutions and MgCl_2_ aqueous solutions were employed as functional liquids while pure water served as the reference condition for configuring four kinds of nanofluidic systems. Pressure-induced percolation experiments were performed on the four zeolite-based systems. The difference in the infiltration process between the electrolyte solution systems and the deionized water system has been ascertained. The effect of the ion size on the infiltration and defiltration process has been determined. The results show that the introduction of ions induces a hydration effect, resulting in a higher critical infiltration pressure of the electrolyte solution system compared to an aqueous solution system. The magnitude of cation charge directly correlates with the strength of the hydration effect and the corresponding increase in critical infiltration pressure. Upon entering the nanochannel, the liquid infiltrates primarily in the form of ions rather than a cation hydration form. The larger the ion size, the shallower the penetration depth after entering the nanopore channel and the larger the corresponding relative outflow rate. The present work will provide valuable theoretical complementary and experimental data support for nanofluidic system applications.

## 1. Introduction

Nowadays, efficient energy conversion and storage is one of the major technological challenges worldwide. Nanofluidic energy absorption system (NEAS), consisting of a suspended nanoporous medium in a non-wetting liquid, exhibits tremendous potential due to its exceptionally high specific surface area. This system offers energy absorption density that is tens or even hundreds of times greater than that of traditional materials [1]. The nanofluidic systems necessitate the application of external pressure to overcome capillary effects and facilitate the intrusion of a nonwetting liquid into energetically unfavorable nanopores. When the external pressure is released, the liquid can be squeezed out, fully or partially, or remain trapped in the solid. Depending on the extent of the liquid outflow, the stored surface tension energy will be fully or partially released, or exclusively absorbed. As a result, the system can behave as a spring or shock absorber or bumper. Based on the energy absorption characteristics of nanofluid systems, high-performance mechanical energy absorption systems of hydrophobic nanopores have been manufactured [2,3]. In this device, when the external pressure is eliminated, the invading liquid can flow out of the nanopore, facilitating the reusability of the energy absorption system [4,5].

The performance of the discussed nanofluidic systems herein is contingent upon the size and type of the nanopore and the type of functional liquid employed. Numerous researchers have analyzed and studied the types and pore sizes of nanoporous media in nanofluidic systems [6,7,8,9,10,11]. At the same time, functional liquids play an influential role as an essential ingredient of the nanofluidic systems. Kong experimentally investigated the effect of addition of ethanol on pressure-induced infiltration of a hydrophobic mesoporous silica [12]. Liu and Cao employed molecular dynamics simulations to analyze the performance of NEAS based on glycerol solutions with varying concentrations. The results show that the energy absorption density of NEAS can be significantly increased by increasing the concentration of glycerol in the liquid environment [13].

The presence of an additive electrolyte can significantly modify the intrusion pressure and transport behaviors of liquid molecules/ions within nanopores, thereby resulting in distinct energy dissipation performances [14]. Both experiment and molecular simulation conducted by Liu demonstrated that smaller ions require a higher pressure to sustain their infiltration into a nanochannel with molecular dimensions [15]. Zhao investigated the leaching process and transport of polar silica nanopores by introducing various electrolytic dissociations into an aqueous solution. The results indicated that the infiltration pressure of nanopore decreases as the ionization scale increases [16]. Gao conducted a systematic study on the outflow process of liquid water and electrolyte solutions from hydrophobic nanopores decorated with grafted silyl chains, proposing nanoconfinement mechanisms involving molecular repulsion/attraction and congestion to achieve a spontaneous outflow with high and stable efficiency, beyond the traditional confinements of hydrophobicity [17]. Han presented the experimental findings on pressure-induced infiltration in the hydrophobic nanopores of a silica gel, where the variation in infiltration pressure remains insignificant across a wide temperature range upon the addition of an electrolyte [18]. To clarify the energy dissipation and conversion mechanisms, experimental infiltration and defiltration tests of liquid/ion solutions into nanopores of a hydrophobic ZSM-5 zeolite were conducted [19]. Lu found that the transport behavior of solvated ions in nanopores of a zeolite Y is responsive to an external electric field. The observed change in the effective solid–liquid interfacial tension contradicts the predictions of classical electrochemical theory; it considerably increases no matter whether the applied voltage is positive or negative [20]. In Kim’s study [21], they found that the anion size has a pronounced influence on the system free energy, thereby affecting the transport process of electrolytes in nanochannels. Cao used molecular dynamics simulations to the transport behavior of a pressure-driven electrolyte solution through a surface-charged nanochannel [22].

The introduction of foreign ions has been shown to strongly influence the liquid intrusion process in NEAS. Due to the distinct behavior of confined liquid molecules/ions at the nanoscale, which is challenging to predict and quantify and still not fully understood, a comprehensive theory cannot be established without conducting more experiments to investigate ion infiltration and transportation in nanopores under diverse conditions.

In the current work, three electrolyte solutions with different ionic sizes were utilized as liquid phase to form NEASs with ZSM-5 zeolite. Pressure-induced infiltration experiments of the electrolyte solution/zeolite systems were conducted. By examining the pressure and volume changes of NEASs during the pressure-induced infiltration process, the motion of pressurized liquid molecules/ions is discussed and the effect of ion size on the infiltration and defiltration process are clarified.

## 2. Experimental Design

The nanoporous material investigated in this study is ZSM-5 zeolite, which was obtained from Shanghai Fuxu Molecular Sieve Co., Ltd. in China. Roasting pretreatment was conducted at 1000 °C for 6 h to improve the purity and stability of the raw material. After the roasting pretreatment, the specific surface area of the ZSM-5 zeolite is 574.68 m^2^/g, the pore volume is 430 mm^3^/g, and the silicon-to-aluminum molar ratio is 173.73. Non-Local Density Functional Theory (DFT) analysis revealed both micro and meso pores in the sample with a mesopore size of 2.214 nm and a micropore size of 0.524 nm.

To gain comprehensive insight into ion effects, functional liquids including KCl aqueous solution, NaCl aqueous solution, MgCl_2_ aqueous solution, and deionized water were used. The electrolyte solution concentration was 4.59 mol∙L^−1^ based on a concentration of saturated KCl aqueous solution at 30 °C. Specified, all percentage concentrations refer to volumetric concentrations in the current work. The pre-treated ZSM-5 zeolite and functional liquid were blended according to 1 g zeolite to 10 mL functional liquid. Subsequently, the mixture was subjected to vacuum degassing treatment for 12 h. After excessive air bubbles introduced during blending were eliminated, the preparation process for nanofluidic stuffing was completed.

**Figure 1:**
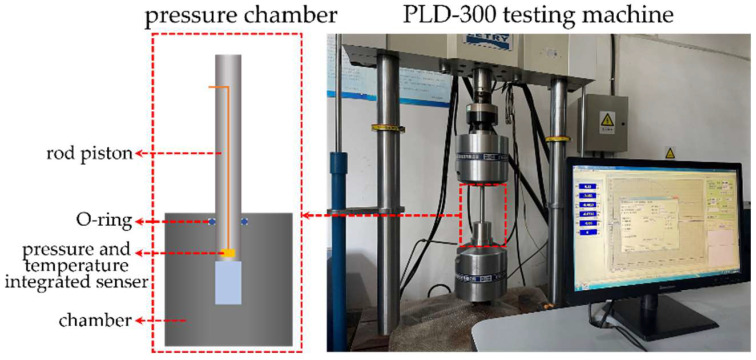
Pressure–volume test bench for NEAS.

The pressure induced infiltration tests were performed on a pressure-specific volume test bench, as depicted in Figure 1, comprising primarily a loading system and a pressure chamber. The loading system employed was a PLD-300 electro-hydraulic servo fatigue testing machine. The pressure–volume test chamber consisted of a chamber and a rod piston, both made of stainless steel. A specially carved O-ring slot was incorporated near the top surface of the pressure chamber to ensure effective sealing between the chamber and the rod piston. Additionally, at the base of the rod piston, an integrated sensor for measuring real-time changes in both pressure and temperature was embedded.

During the experiments, the degassed nanofluidic stuffing should be placed in the pressure chamber. Subsequently, the rod piston was driven into the chamber by means of the PLD-300 testing machine at a loading rate of 0.02 mm∙s^−1^. Once the pressure in the chamber reached approximately 50 MPa, the rod piston was retracted at an identical speed, until it returns to its initial position, completing a loading–unloading loop. In the current study, ten consecutive loading–unloading loops were conducted.

## 3. Result and Discussion

### 3.1. Pressure-Specific Volume Curves of Electrolyte Solution/Zeolite System

**Figure 2:**
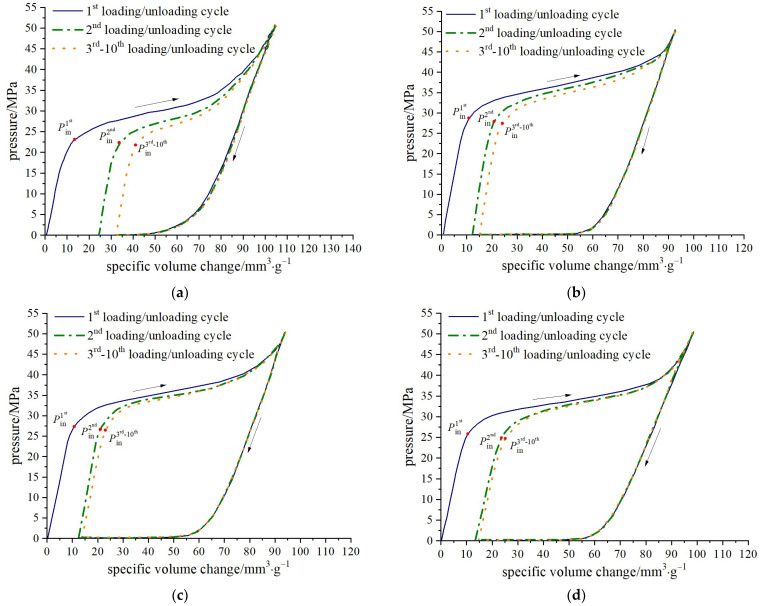
Pressure-specific volume curves of water/zeolite, KCl solution/zeolite, NaCl solution/zeolite, and MgCl_2_ solution/zeolite (zeolite here refers to ZSM-5 zeolite, where zeolite and the functional liquid are blended according to 1 g to 10 mL, the concentration for the three electrolyte solution are 4.59 mol∙L^−1^, and the loading rate is 0.02 mm∙s^−1^): (**a**) water/zeolite; (**b**) KCl solution/zeolite; (**c**) NaCl solution/zeolite; and (**d**) MgCl_2_ solution/zeolite.

The pressure-specific volume curves for the four zeolite-based nanofluidic systems are presented in Figure 2, namely water/zeolite, KCl solution/zeolite, NaCl solution/zeolite and MgCl_2_ solution/zeolite systems. Pressure is monitored with the pressure sensor embedded in the rod piston. The specific volume change is defined as the space occupied by the piston normalized by the mass of ZSM-5 zeolite; that is, Δ*V* = *A*Δ*d*/*m* where *A* is the cross-sectional area of the stainless-steel rod piston, Δ*d* is the rod piston’s displacement, and *m* is the mass of zeolite.

The characteristic features of a typical NEAS percolation process can be identified from the pressure-specific volume curves. During the loading period, as the piston moves downwards, the system volume is compressed and the internal pressure increases. Once the pressure reaches a critical value (*P*_in_), capillary repulsion is overcome and pressure-induced infiltration occurs, resulting in an abrupt decrease in the sorption curve slope and the formation of a plateau region. Eventually, when most accessible nanopores are filled, the plateau region ends, and system compressibility decreases significantly. At around 50 MPa, loading ceases and the unloading begins with functional liquid being expelled from the nanochannels due to solid phase lyophobicity.

### 3.2. Effect of Ion Size on Critical Infiltration Pressure

**Figure 3:**
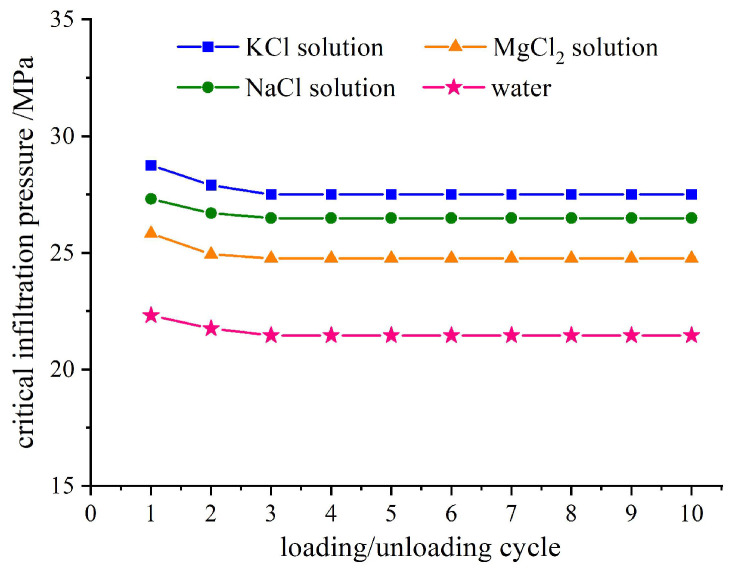
Effect of ion species on critical infiltration pressure under ten cyclic loadings(NEASs tested are water/zeolite, KCl solution/zeolite, NaCl solution/zeolite, and MgCl_2_ solution/zeolite; loading rate is 0.02 mm/s).

The infiltration pressure of ten loading–unloading loops in the four studied systems is depicted in Figure 3. It should be noted that aqueous electrolytes exhibit higher surface tensions compared to pure water [23]. Consequently, the infiltration pressure *P*_in_ = 4*γ*/*d* [24] will inevitably increase due to the enhanced surface tension of the aqueous electrolytes.

In addition, the infiltration pressure of the system is comparable to the ionic radius of the three different cations at the same cation concentration. The crystal ion radii values for potassium, sodium, and magnesium in their common valence state are potassium ion K^+^: 152 pm, sodium ion Na^+^: 116 pm, and magnesium ion Mg^2+^: 71 pm [25]. When cation dissolves into an aqueous solvation, water molecules cluster around it. The negatively charged oxygen atoms in water are attracted to the positive charge of the metal cations, forming a hydration layer with a thickness equivalent to a few molecular sizes. The thickness of this hydration layer depends on the amount of cation charge [26]. Consequently, the apparent diameter of the molecules/ions of the functional liquid is equivalently increased [27,28,29], which inevitably hinders the infiltration process. This ultimately results in higher infiltration pressure for electrolytic solution and requires more external mechanical work during liquid phase intrusion into nanopores. However, the cations will first dissociate from water molecules that are aggregated due to cation hydrodynamics and then enter the channels as ions (this will be discussed in detail in Section 3.3). The impact of valence of the cations on infiltration pressure is negligible due to the minimal force required for intermolecular fracture of the hydrated layer. The infiltration pressure is in accordance with the order of the ion size. In subsequent loading processes (second and third loops), the order of infiltration pressure for different cation solution systems aligns with that based on cation size following the aforementioned aggregation effect rule. The infiltration pressure for successive loops is slightly lower than that observed in the first loop.

### 3.3. Effect of Ion Size on Accessible Pore Volume

**Figure 4:**
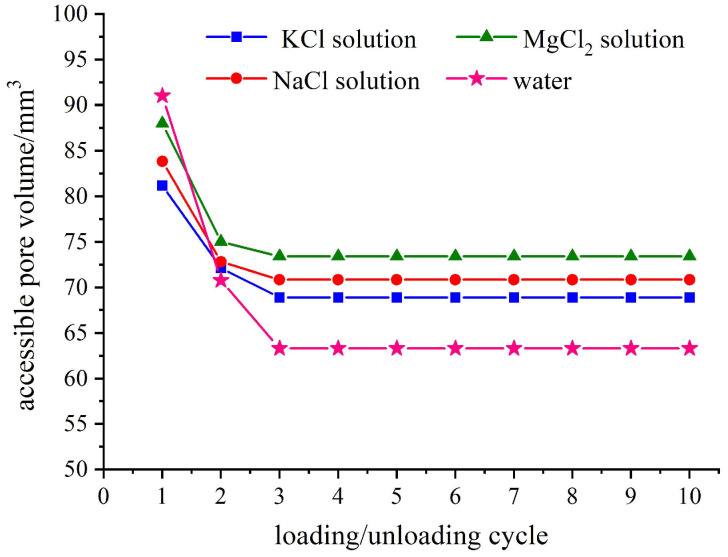
Effect of ion species on accessible pore volume under ten cyclic loadings(NEASs tested are water/zeolite, KCl solution/zeolite, NaCl solution/zeolite, and MgCl_2_ solution/zeolite; loading rate is 0.02 mm/s).

The hydration of cations in an electrolyte solution not only results in a higher infiltration pressure upon fluid entry into the nanopore, but also restricts the specific capacity of the nanopore to accommodate additional liquid molecules/ions. Figure 4 illustrates the accessible pore volumes for four types of zeolite-based systems with ten loading–unloading loops. It can be observed from the figure that the water/zeolite system exhibit the maximum accessible pore volume during the first loading–unloading loop, but experiences a significant decrease in subsequent loops compared to the electrolyte solution/zeolite systems. This phenomenon is attributed to the incomplete drainage of the infiltrated liquid from zeolite pores after the initial loading–unloading loop, which consequently occupies an effective inflow space for subsequent loops. This aspect will be thoroughly examined in the following section, along with data on relative outflow rates.

The accessible pore volume of the electrolyte solution/zeolite system exhibits a consistent trend in each loop. Specifically, as the cation size increases, the accessible pore volume decreases. Zeolites possess a certain distribution of pore sizes, and when the liquid type is altered, changes in cations size render some previous accessible pores inaccessible. Furthermore, Figure 4 demonstrates that the infiltration depth of liquid molecules within nanochannels varies based on the value of accessible pore volume. In systems with large ionic size, the infiltration depth of liquid molecules/ions is shallower. Among all systems examined, it is observed that the KCl solution/zeolite system corresponds to both the smallest accessible pore volume and the shallowest infiltration depth.

Considering the effect of cation hydration, there are two potential scenarios regarding the mechanism by which liquid molecules/ions in electrolyte solutions enter the nanochannels. One possibility is that the cations will first dissociate from water molecules that are aggregated due to cation hydrodynamics and then enter the channels as ions. The other possibility is that the cations will enter the channel along with previously hydrated water molecules. Analyzing and comparing the infiltration process between the MgCl_2_ solution/zeolite system and KCl solution/zeolite system or NaCl solution/zeolite system can provide insights into understanding the infiltration process of electrolyte solutions. Since Mg^2+^ ion carries two positive charges, it tends to aggregate a greater number of layers of water molecules through hydration compared to K^+^ and Na^+^. If the cations enter the pore accompanied by water molecules aggregated via cation hydration, then we would expect that the infiltration pressure in MgCl_2_ solution/zeolite system should be higher than those in other electrolyte solutions/zeolite systems. However, contrary to this expectation, it is actually lower than both KCl and NaCl solutions, only slightly higher than pure water as depicted in Figure 3. Therefore, it can be confidently concluded that our initial assumption holds true: The cations primarily detach from water molecules aggregated via hydration and enter the nanochannel as ions. Furthermore, once inside these nanochannels, the depth of liquid molecules in the MgCl_2_ solution/zeolite system is also found to be the deepest among all three systems studied so far; this further supports our conclusion that the size of the cation plays a decisive role in the infiltration depth when the amount of charge is different.

### 3.4. Effect of Ion Size on Relative Outflow Rate

After the first loading/unloading loop of the NEASs in this study, the unloading pressure-specific volume characteristic curve does not return to its initial point when the pressure returns to 0.1 MPa. This suggests that the porous material in the system may have undergone permanent structural collapse damage due to compressive loading or that liquid molecules/ions infiltrating into a state cannot flow out and remain trapped within pores. It is generally believed that both causes contribute to volume loss after the first loading–unloading loop, while subsequent loops’ volume loss is only related to liquid molecules/ions retention.

**Figure 5:**
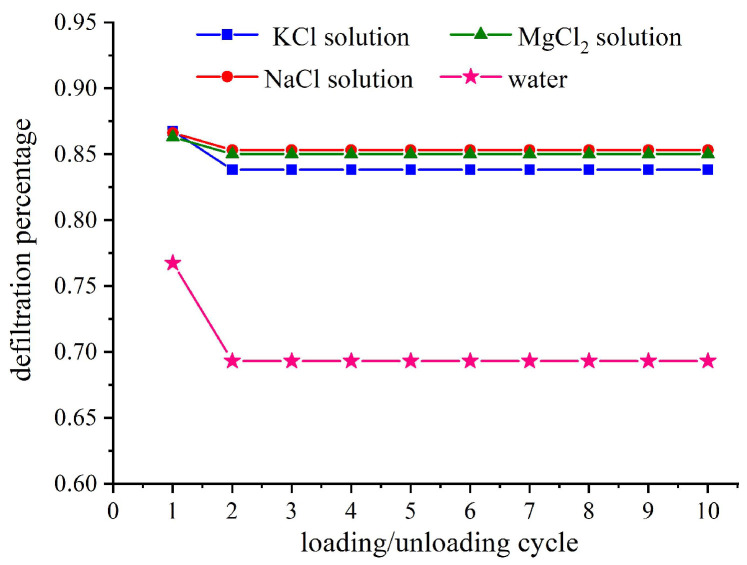
Effect of ion species on defiltration percentage under ten cyclic loadings (NEASs tested are water/zeolite, KCl solution/zeolite, NaCl solution/zeolite, and MgCl_2_ solution/zeolite; loading rate is 0.02 mm/s).

The relative outflow rate is defined as the ratio of the liquid volume flowing out of the porous material during the current cycle to the volume of liquid flowing into the porous material during the initial loading process, reflecting the extent to which liquid flows out of the nanochannel after unloading. Figure 5 illustrates the relative outflow rates of the studied NEASs after ten loading–unloading loops. As can be seen from the figure, compared to the water/zeolite system, the electrolyte solution/zeolite system exhibits a higher relative outflow rate, while ionic size has minimal impact on this parameter. By comparing with Figure 4, it can be observed that deeper intrusion leads to increased trapping probability and subsequently lower relative outflow rate.

When the liquid and porous material are confined in a closed system to form the NEAS, the liquid’s infiltration depth is determined not only by the structure of the channel itself, but also by two factors: one is the nature of the liquid itself, and the other is the state of the gas (air) remaining in the channel. Prior degassing of the mixed sample cannot eliminate the initially trapped air within the porous material. During experimental pressurization, as liquid intrudes into the channel, air will be continuously squeezed, with most forming clusters within it, while a small fraction diffuses out through fluid molecules. The extent of diffusion depends on properties specific to each liquid. As fluid flows into channels within porous materials, repulsive forces from solid walls prevent complete contact between fluid molecules and the walls; instead, an equilibrium distance is established. Reference [16] studied the confinement behavior of different electrolyte solutions in carbon nanotubes using numerical simulation. It revealed that larger ionic sizes result in smaller equilibrium distances between fluid molecules and solid walls. This equilibrium distance provides a circular path for the leakage of gas molecules. Consequently, smaller ion sizes lead to larger equilibrium distances allowing more gas molecules to diffuse out from channels thereby creating additional space for liquid molecules/ions and increasing the accessible pore volume. Furthermore, this equilibrium distance determines frictional dissipation experienced by liquid during their passage through channels and affects penetration depth of the liquid molecule/ion into holes. The smaller the equilibrium distance is, the closer the liquid molecules/ions are to the channel wall, resulting in an increased effective shear stress and a greater friction dissipation. This indicates that all energy is consumed during infiltration, leading to a shallower depth of fluid molecules into the nanochannel.

More importantly, cation exchange takes place within the nanochannel as cations diffuse into the zeolite framework. The nanopore surface may exhibit reduced polarization due to pre-existing negatively charged surface sites, such as hydroxyl groups or other defects, which can be counterbalanced by cations. Consequently, the effective number density of surface defects decreases, rendering the nanopore surface more lyophobicity and less attractive to water molecules [30], thereby enhancing the relative outflow rate.

## 4. Conclusions

In the present study, NEASs were configured with the ZSM-5 zeolite as porous medium and deionized water, KCl solution, NaCl solution and MgCl_2_ solution as functional liquids. The pressure-induced infiltration experiment was conducted on the pressure–volume test bench. The influence of ion size on the liquid infiltration and defiltration process in NEASs is discussed. The results indicate that the infiltration pressure of the system is comparable to the ionic radius of the three different cations. Hydration effects result in the formation of a hydration layer around the cation, leading to higher infiltration pressure for electrolytic solutions and increased external mechanical work during liquid molecules/ions intrusion into the nanopores.

For the infiltration process, the accessible pore volume of the electrolyte solution/zeolite system is significantly lower compared to that of the deionized water/zeolite system. The larger the cation size, the smaller the accessible pore volume becomes. By comparing the infiltration process between the MgCl_2_ solution/zeolite system and the KCl or NaCl solution/zeolite system, it can be demonstrated that the cations enter the nanochannels as ions instead of a cation hydration form. The ion size influences both the equilibrium distance and polarity of the nanochannel, thereby affecting the relative outflow rate during the defiltration process. Relative outflow rate of the electrolyte solution/zeolite system is higher than that of the water/zeolite system, with ionic size having a slight impact on the relative outflow rate.

## Data Availability

Not applicable.
